# Cutaneous vasculitis in patients with autoimmune polyendocrine syndrome type 1: report of a case and brief review of the literature

**DOI:** 10.1186/1471-2431-14-272

**Published:** 2014-11-01

**Authors:** Nicola Improda, Donatella Capalbo, Emilia Cirillo, Manuela Cerbone, Andrea Esposito, Claudio Pignata, Mariacarolina Salerno

**Affiliations:** Unit of Pediatric Endocrinology, Department of Traslational Medical Sciences, “Federico II” University of Naples, Naples, Italy; Unit of Pediatric Immunology, Department of Traslational Medical Sciences, “Federico II” University of Naples, Naples, Italy

**Keywords:** APS 1, *AIRE*, Cutaneous vasculitis, Autoimmunity

## Abstract

**Background:**

Autoimmune polyendocrine syndrome type 1, also known as autoimmune polyendocrinopathy-candidiasis-ectodermal-dystrophy, is a rare autosomal recessive disease due to pathogenic variants in the *AIRE* gene. Classic features of the syndrome are mucocutaneous candidiasis, chronic idiopathic hypoparathyroidism and Addison disease. However, other endocrine and non-endocrine components, may occur with a different prevalence. In addition to ectodermal features, which are quite common features of the disease, APS 1 patients may experience other types of skin alterations, such as vasculitic skin rash. An early diagnosis of APS 1 can be very challenging, due to the high clinical heterogeneity, and a considerable delay may occur between the appearance of symptoms and the diagnosis.

**Case presentation:**

We report on a girl affected by APS 1 who presented with cutaneous vasculitis when she was seven-months old, some years before the onset of the common components of the disease.

**Conclusion:**

Clinical picture of APS 1 may be characterized by isolated rare or atypical autoimmune or immune-mediated manifestations, even years before the onset of the classic components of the disease. Among these uncommon features, skin rashes of variable form and duration may occur, most of them being associated with histopathological features of vasculitis. Our case suggests that cutaneous vasculitis may represent a first sign of APS 1. The clinical significance of cutaneous vasculitis in the context of APS 1 is still debated. It may represent a rare, unusual, early component of the disease or a clinical manifestation secondarily related to the typical APS 1 components (i.e. autoimmune thyroid disease), which are frequently associated with rheumatologic-like signs and symptoms. Alternatively, it may be the expression of an independent disease co-occuring with APS 1. In conclusion, our case suggests that children presenting with unexplained vasculitic skin rash should be followed-up in order to early identify APS 1.

## Background

Autoimmune polyendocrine syndrome type 1 (APS 1), also known as autoimmune polyendocrinopathy-candidiasis-ectodermal dystrophy (APECED), is a rare autosomal recessive disease caused by pathogenic variants in the autoimmune regulator (*AIRE*) gene. *AIRE* encodes for the homonymous protein, AIRE, which acts as a regulator of the process of gene transcription and is involved in the mechanisms of deletional central (and presumably peripheral) tolerance. AIRE deficiency leads to the escape and extra-thymic spreading of autoreactive T-cell clones: this creates the basis for the onset of the autoimmune attack against several tissue-specific self-antigens [1].

The clinical diagnosis of APS 1 is defined by the presence of at least two components of the classic triad, which is given by chronic mucocutaneous candidiasis (CMC), chronic idiopathic hypoparathyroidism (HPT) and Addison disease (AD). The disease generally begins in childhood and CMC is the first component appearing by five years of age, followed by HPT and then by AD. Other endocrine and non-endocrine components, such as hypergonadotropic hypogonadism, hypothyroidism, type 1 diabetes, gastrointestinal dysfunction, autoimmune hepatitis, asplenia and various ectodermal abnormalities (interstitial keratitis, alopecia, vitiligo, nail dystrophy and dental enamel hypoplasia), may occur with a different prevalence [2–5]. In addition to ectodermal features, which are quite common features of the disease, APS 1 patients may experience other types of skin alterations. Indeed, in a restricted number of cases a maculopapular, or morbilliform, or urticaria-like skin rash, eventually accompanied by fever, splenomegaly and arthralgia, has been reported [2, 3, 6–18]. When performed, biopsy of the above lesions has revealed perivascular, lymphoplasmacytic infiltrates in most of the cases [3, 11, 12, 18]. Whether skin involvement represents the expression of a direct autoimmune attack, or an unrelated event still remains to be defined.

Here we report on a challenging diagnosis of APS 1 in a patient who presented at a very early age with a urticarial skin rash, with histopathological evidence of vasculitis at skin biopsy, some years before the onset of other classic components of the disease.

## Case presentation

A 7-month-old female of non-consanguineous parents, presented with a skin rash consisting of purple plaques (maximum diameter 4 cm) with irregular and erythematous margins, which were localized to the trunk and limbs. The child also had mild splenomegaly and relapsing episodes of joint pain with fever. Skin biopsy showed inflammatory infiltrates within and around the walls of small vessels with signs of endothelial damage in the form of endothelial swelling, thus confirming a diagnosis of vasculitis. The child underwent a diagnostic work-up, which showed increased levels of C-reactive protein (27 mg/dl; n.v. <0.5), erythrocyte sedimentation rate (66 mm/hour; n.v. <10) and immunoglobulins (IgG 30.9 g/l; n.v. 1.7-10.7 and IgM 1.63 g/l; n.v. 0.3-1.3). C3 and C4 complement factors were within the normal range (C3 1.13 g/l; n.v. 0.6-1.8 and C4 0.7 g/l; n.v. 0.07-0.7). Antibodies against common infectious agents were negative. The percentage of double negative T lymphocytes (CD3 + CD4-CD8-), the lymphocyte response to mitogens and lymphocyte sensitivity to FAS-induced apoptosis were all normal. Anti-nuclear (ANA), perinuclear (p-) and cytoplasmic (c-) anti-neutrophil cytoplasmic (ANCA), anti-thyroid, anti-double stranded (DS) DNA, anti-phospolipids antibodies were all undetectable. Skin lesions regressed spontaneously by the second year of life; biochemical abnormalities also normalized during the follow-up except levels of IgG and IgM which remained elevated. The patient was then referred at the age of 5 years because of recurrent oral candidiasis, alopecia of eyelashes and eyebrows, autoimmune thyroiditis, abdominal pain and diarrhea. Despite a mild increase in serum TSH levels (6 mIU/l), free-T4 (FT4) levels were normal, and the patient did not require levothyroxine treatment [19].

Based on the persistence of candidiasis, cutaneous manifestations and autoimmune thyroiditis, direct sequencing of the *AIRE* gene was performed, revealing 47C > T and 232 T > A variants in the exons 1 and 2, respectively, thus confirming the diagnosis of APS 1. These variants were inherited from the parents. Autoantibodies evaluation revealed positive anti-thyroid, adrenal cortex, 17- and 21-hydroxylase, gastric parietal cells, tryptophan hydroxylase, side-chain cleavage, L-amino acid decarboxylase, and steroid-producing cells antibodies (Table 1). Antibodies against the IA-2 tyrosine phosphatase-like protein, insulin, and glutamic acid decarboxylase were negative on several controls. Anti-interferon antibodies were first evaluated when she was 9 years-old, and were found to be positive.

**Table 1:** **First detection of specific autoantibodies compared to the age of onset of APS 1 components**

Specific autoantibodies	Age at first test (years)	Age at first detection (years)	Related APS 1 component	Onset of APS 1 component (years)
Anti-thyroglobulin	5	5	Autoimmune thyroiditis	5
Anti-thyreoperoxidase	5	5	Autoimmune thyroiditis	5
Anti-parietal cells	7	7	Autoimmune gastritis	-
Anti-adrenal cortex	7	7	Addison disease	11
Anti-21-hydroxylase	8	8	Addison disease	11
Anti-17alpha hydroxylase	8	8	Addison disease	11
			Ovarian failure	12
Anti-P450 side chain cleavage	8	8	Addison disease	11
			Ovarian failure	12
Anti-steroid-producing cells	8	8	Ovarian failure	12
Anti-tryptophan hydroxylase	9	10	Autoimmune hepatitis	-
			Autoimmune enteropathy	5
Anti-L-aminoacid decarboxylase	9	10	Vitiligo	9
			Autoimmune hepatitis	-

During the follow-up the patient developed other signs and symptoms of the disease. At the age of 9 years, she was noted to have areas of vitiligo and signs of ectodermal dystrophy, such as dental enamel and nail dysplasia. HPT was diagnosed at the age of 9 years on the basis of low calcium (1.5 mmol/l) and parathyroid hormone levels (7 pg/ml, n.v. 10–65), and a treatment with calcitriol and calcium supplementation was started. At the age of 11 years, increased levels of ACTH (150 pg/ml; n.v. 10–130) and renin (184 pg/ml; n.v. 1.8-3.3 pg/ml), with reduced cortisol peak (108 ng/ml) after ACTH stimulation test, associated with presence of anti-adrenal cortex, 17- and 21-hydroxylase antibodies, led to the diagnosis of AD; therefore, glucocorticoid and mineralcorticoid replacement therapy were started. Six months later she also started levothyroxine treatment due to a further increase in TSH values (TSH 15 mU/ml), with reduced values of FT4 (0.6 ng/dl; n.v. 0.9-1.7). Patient’s pubertal development was normal and she experienced menarche at the age of 10 years. However, at the age of 12 she had secondary amenorrhea with increased gonadotropin levels (FSH 50 mUI/ml; LH 35 mUI/ml). According to positivity of anti-steroid-producing cells antibodies, a diagnosis of premature ovarian failure due to autoimmune oophoritis was made, therefore she started hormonal replacement therapy with estrogens and progestins.

The clinical follow-up was also marked by an increase in the extent of the alopecia, which affected over time a large part of the scalp. Table 2 summarizes the clinical components occurring over time in our patient, according to the age of onset.

**Table 2:** **Clinical course of APS 1 in our patient**

APS 1 component	Age of onset (years)
Cutaneous vasculitis	0.7
Mucocutaneous candidiasis	2
Abdominal pain with stipsis and diarrhea	5
Autoimmune thyroiditis	5
Alopecia	5
Ectodermal dystrophy	9
Vitiligo	9
Chronic idiopathic hypoparathyroidism	9
Addison disease	11
Ovarian failure	12

## Discussion

We report on a child with APS 1 who presented with cutaneous vasculitis at a very early age, more than one year before the onset of the typical features of the disease.

Some clinical aspects and genetics of the patient described have been already included in a small case series described by Capalbo et al. [20] and within an Italian case series described by Mazza et al. [4]. However, these two papers focused on the genetics and the phenotypic heterogeneity of APS 1 patients originating from the same geographic area (the former) and from several Italian regions (the latter), and the clinical counterpart of our patient was only briefly and partially discussed.

Aim of the current paper was to describe in detail this patient with unusual presentation of APS 1, and to report information of its clinical course. Indeed, in our opinion such case raises interesting issues regarding the eventual involvement of the skin in APS 1 and aware physicians to consider the diagnosis of APS 1 when evaluating a patients with unexplained cutaneous vasculitis.

APS 1 is characterized by high phenotypic heterogeneity, with a great variability in the number of the clinical manifestations and the age at onset of the disease even between patients with the same genotype [3–5]. Indeed, the first clinical signs and/or symptoms may occur from the first months of life up to adulthood [21], the earlier presentation being generally associated with a more severe phenotype and a higher number of clinical components [1]. Although in its typical form APS 1 presents with at least one of the classic triad, some patients suffer from several minor manifestations of the disease for many years before one of the first major components occurs [2].

When a rare or atypical component is the presenting feature of the disease, the diagnosis of APS 1 can be challenging and a considerable delay may occur between the appearance of symptoms and the diagnosis [22].

Although skin rash represents a rare manifestation of APS 1, to date a few patients with different forms of cutaneous eruptions have been described (Table 3) [2, 3, 6–18]. Only the minority of the cases reported were less than 1 year old [3, 8, 15].

**Table 3:** **Previous reports of skin involvement in APS 1 patients**

Author and year of publication	N. of cases	Age at the onset of skin lesions (yrs)	Aspect of skin lesions	First typical component, age at onset (yrs)	Biopsy
**Case series**					
Craig JM et al. 1955 [7]	1/3	2.8	erythema marginatum/recurrent skin rash	CMC, 3	increased melanin content
Betterle C et al. 1998 [2]	1/41	n.a.	n.a.	n.a.	n.a.
Perheentupa J 2006 [3]	13/91	0.7–31	fleeting maculopapular, morbilliform, or urticarial rash	n.a	2/4 biopsies revealed vasculitis
Trebušak Podkrajšek K et al. 2008 [6]	1/11	2	n.a.	HPT, 7.5	n.a.
Posovszky C et al. 2012 [15]	2/13	1	n.a.	CMC, 2.0	n.a
		5	chronic recurrent urticaria	CMC, 3.0	
**Case reports**					
Quinto MG et al. 1964 [8]	1	0.9	multiform erythema	HPT + CMC, 4.0	n.a.
Stickler GB et al. 1965 [9]	1	7.6	evanescent trunkal macular rash	HPT + CMC, 9.1	n.a.
Spörkmann K-H et al. 1990 [10]	1	1.2	multiform erythema	HPT, 3.0	n.a.
Garty B 1998 [11]	1	22	erythema annulare centrifugum	HPT, 5	lymphohistiocytic vasculitis
Füchtenbusch M et al. 2003 [12]	1	31	purpuric subepidermal nodules progressing in deep cutaneous ulcers	HPT, 3.0	panniculitis and lymphocytic vasculitis
Kapelari K et al. 2004 [13]	1	16	photosensitive facial rash (diagnosis of systemic lupus erythematosus)	n.a.	n.a.
Hoorweg-Nijman G et al. 2008 [14]	1	1.2	n.a.	HPT, 9.0	n.a.
Montin D et al. 2008 [16]	2	1.1	urticarial rash (vasculitic rash)	CMC, 1.5	n.a.
		7	urticarial rash	CMC, 7.0	
Rodríguez Sánchez De La Blanca A et al. 2012 [18]	1	6	photosensitive rash	HPT, 9.0	n.a.
O’ Gorman CS et al. 2013 [17]	1	1	intermittent urticarial rash	HPT + CMC, 1	lymphocytic vasculitis

CMC: chronic mucocutaneous candidiasias; HPT: chronic idiopathic hypoparathyroidism; n.a.: not available.

Reports from Finnish population showed that 14% (13 out of 91 patients) of their cohort may exhibit periodic morbilliform, maculopapular or urticarial skin rashes with fever and/or arthralgia, appearing at age 0.7-31 years and lasting for 0.2-1.2 yr. Five out of these 13 patients, aged 0.7-1.2 years, showed high plasma IgG levels. Moreover, four patients underwent skin biopsy, revealing a lymphoplasmacytic vasculitis in two and no specific pathology in the other cases. The same author hypothesized an autoimmune pathogenesis of such component [3].

In another large series of 41 APS 1 subjects from Italy, only one patient was described as having cutaneous vasculitis [2].

In a cohort of 11 APS 1 patients from different European countries (Serbia, Slovenia and Germany) the authors described a Serbian APS 1 girl first presenting with recurrent episodes of high fever, accompanied by cutaneous rash and arthralgias at the age of two years, who was diagnosed with systemic juvenile rheumatoid arthritis. This patient also suffered from asthma-like dyspnea and developed the first major APS 1 component (HPT) only when she was 7.5 years old [6].

Skin biopsy was performed in only a few cases, revealing in most, but not all, evidence of an underlying vasculitis [3, 11, 12, 18]. For this reason, the prevalence of cutaneous vasculitis in the context of APS 1 is unknown and it might to be higher than that reported so far. As for our patient, vasculitic skin rashes in patients with APS 1 have been previously reported to be associated with other signs and/or symptoms, such as therapy-resistant and/or recurrent fever [6, 7, 9, 10, 13–16, 18], polyarthritis [15], arthralgia [3, 6, 12], hepato-splenomegaly [7, 16] and photosensitivity [13, 17], as well as with laboratory and/or histological abnormalities such as hypergammaglobulinemia [9, 13, 16], elevated erythrocyte sedimentation rate [9, 13], positive rheumatoid factor [15], traces of cryoglobulinemia [2] and panniculitis [12].

The clinical significance of cutaneous vasculitis in the context of APS 1 is still debated. It may represent a rare, unusual, early component of the disease or a clinical manifestation secondarily related to the typical APS 1 components (i.e. autoimmune thyroid disease), which are frequently associated with rheumatologic-like signs and symptoms. Alternatively, it may be the expression of an independent disease co-occuring with APS 1.

In this regard, taking into account that a complex clinical picture including skin rash may continue for years before some of the classic APS 1 components appear, it is not surprising that some patients have been initially suspected or diagnosed as having rheumatologic diseases like juvenile rheumatoid arthritis and Wissler-Fanconi syndrome [6, 10] or other autoimmune disorders such as autoimmune hepatitis [15]. In our patient, the presence of early-onset cutaneous vasculitis, mild splenomegalia, and serum hypergammaglobulinemia first suggested a diagnosis of autoimmune lymphoproliferative syndrome (ALPS). ALPS is a chronic, non-malignant lymphoproliferative disorder due to mutations in the genes involved in apoptosis. As for APS 1, it presents in the first years of life (usually by 5 years of age) and its natural history is characterized by the development of multiple autoimmune manifestations [23]. However, in our case the normal lymphocyte sensitivity to FAS-induced apoptosis and the absence of double negative T cells (CD3 + CD4-CD8-), which represent the immunological hallmark of the disease, definitely ruled out a diagnosis of ALPS. The diagnosis of APS 1 was suspected only when recurrent oral candidiasis and several autoimmune components became evident, some years after the onset of cutaneous vasculitis.

Early diagnosis of APS 1 and ongoing regular surveillance, including periodic evaluation of hormonal and biochemical parameters, are essential to allow the prevention of severe and life-threatening events (i.e. hypocalcaemia, adrenal crisis) [2]. Therefore, although clinical components of APS 1 usually result from organ-specific autoimmune targeting, our case suggests that APS 1 should be also suspected in those cases presenting with immune-mediated non-organ-specific diseases, such as cutaneous vasculitis.

Recently, neutralizing autoantibodies against type 1 interferons (IFN) (IFN-α and IFN-ω) have been found to strictly correlate with *AIRE* deficiency, regardless of the genotype, thus leading to consider these autoantibodies as a precocious diagnostic tool for APS 1, even in the absence of the typical clinical picture or organ-specific autoantibodies [24, 25]. However, it must be considered that molecular analysis, despite more expensive than autoantibody assay, may be more easily accessible for many laboratories. In our case, assay for anti-IFN antibodies was not available at the onset of the disease in our patient, but the above antibodies were found to be positive at the age of 9 years. Therefore, we can only speculate that their positivity could have been useful for an earlier diagnosis.

In addition, our case confirms the importance of the surveillance in searching for other sentinel autoantibodies, mainly those against the adrenal cortex and the related antigen targets, which show high predictive value for the occurrence of the related clinical component [26, 27].

## Conclusions

In conclusion, although causal relationship between APS 1 and skin rashes or cutaneous vasculitis is still unclear, there is evidence pointing toward a close link between these conditions. Based on this hypothesis, our case provides further evidence that several minor or rare autoimmune or immune-mediated organ- and non-organ-specific diseases, such as cutaneous vasculitis, may dominate the initial clinical picture of APS 1, even for years before the development of the classic components of the disease. Therefore, although the diagnostic criteria of APS 1 remain valid, an atypical phenotype of APS 1 should be suspected in the presence of an early non-specific immune-mediated manifestation. Given the high specificity for APS 1 of anti-IFN autoantibodies, their evaluation may be a simple diagnostic tool for an early diagnosis. Finally, a regular check for hormonal, biochemical abnormalities, and organ-specific antibodies should be performed during follow-up, in order to recognize immune-mediated organs damage at an early stage, thus allowing to prevent potentially life-threatening events, such as hypocalcemia and adrenal failure.

## Consent

Written informed consent was obtained from the patient’s parents for publication of this Case report. A copy of the written consent is available for review by the Editor of this journal.

## Abbreviations

APS 1: Autoimmune polyendocrine syndrome type 1
APECED: Autoimmune polyendocrinopathy candidiasis-ectodermal-dystrophy
*AIRE*: Autoimmune regulator gene
CMC: Chronic mucocoutaneous candidiasis
HPT: Chronic idiopathic hypoparathyroidism
AD: Addison disease
ANA: Anti-nuclear antibodies
p- and c- ANCA: Perinuclear and cytoplasmic anti-neutrophil cytoplasmic antibodies
FT4: Free-T4
ALPS: Autoimmune lymphoproliferative syndrome
IFN: Interferons.

## References

[1] Capalbo D, Giardino G, De Martino L, Palamaro L, Romano R, Gallo V, Cirillo E, Salerno M, Pignata C (2012). Genetic basis of altered central tolerance and autoimmune diseases: a lesson from AIRE mutations. Int Rev Immunol.

[2] Betterle C, Greggio NA, Volpato M (1998). Clinical review 03: Autoimmune polyglandular syndrome type 1. J Clin Endocrinol Metab.

[3] Perheentupa J (2006). Autoimmune polyendocrinopathy-candidiasis-ectodermal-dystrophy. J Clin Endocrinol Metab.

[4] Mazza C, Buzi F, Ortolani F, Vitali A, Notarangelo LD, Weber G, Bacchetta R, Soresina A, Lougaris V, Greggio NA, Taddio A, Pasic S, de Vroede M, Pac M, Kilic SS, Ozden S, Rusconi R, Martino S, Capalbo D, Salerno M, Pignata C, Radetti G, Maggiore G, Plebani A, Notarangelo LD, Badolato R (2011). Clinical Heterogeneity and diagnostic delay of autoimmune polye ndocrinopathy-candidiasis-ectodermal distrophy syndrome. Clin Immunol.

[5] Capalbo D, Fusco A, Aloj G, Improda N, Vitiello L, Dianziani U, Betterle C, Salerno M, Pignata C (2012). High intrafamilial variability in autoimmune polyendocrinopathyectodermal-distrophy: a case study. J Endocrinol Invest.

[6] Trebušak Podkrajšek K, Milenković T, Odink RJ, Claasen-van der Grinten HL, Bratanić N, Hovnik T, Battelino T (2008). Detection of a complete autoimmune regulator gene deletion and two additional novel mutations in a cohort of patients with atypical phenotypic variants of autoimmune polyglandular syndrome type 1. Eur J Endocrinol.

[7] Craig JM, Schiff LH, Boone JE (1955). Chronic moniliasis associated with Addison’s disease. Am J Dis Child.

[8] Quinto MG, Leikin SL, Hung W (1964). Pernicious anemia in young girl associated with hypoparathyroidism, familial Addison’s disease, and moniliasis. J Pediatr.

[9] Stickler GB, Peyla TL, Dower JC, Logan GB (1965). Moniliasis, steatorrhea, diabetes mellitus, cirrhosis, gallstones, and hypoparathyroidism in a 10-year-old boy. Clin Pediatr.

[10] Spörkmann K-H, Eickoff R, Dominick HC (1990). Subsepsis allergica bei einer Patientin mit polyglandulärem Autoimmunesyndrom Typ I. Monatsschr Kinderheilkd.

[11] Garty B (1998). Erythema annulare centrifugum in a patient with polyglandular autoimmune disease type 1. Cutis.

[12] Füchtenbusch M, Vogel A, Achenbach P, Gummer M, Ziegler AG, Albert E, Standl E, Manns MP (2003). Lupus-like panniculitis in a patient with autoimmune polyendocrinopathy-candidiasis-ectodermal dystrophy (APECED). Exp Clin Endocrinol Diabetes.

[13] Kapelari K, Raber G, Steinmayr-Gensluckner M, Schweitzer K, Schirmer M, Hoegler W, Zimmerhackl LB (2004). Systemic lupus erythematosus (SLE) in a 14 year-old girl with autoimmune polyendocrinopathy syndrome type 1 (APS-1) [abstract]. Horm Res.

[14] Hoorweg-Nijman G, van Wieringen H, de Vroede M (2008). Heterogeneous presentation and clinical course in two brothers with autoimmune polyendocrinopathy 1 (APECED) due to compound heterozygote mutations in the AIRE gene [abstract]. Horm Res.

[15] Posovszky C, Lahr G, von Schnurbein J, Buderus S, Findeisen A, Schröder C, Schütz C, Schulz A, Debatin KM, Wabitsch M, Barth TF (2012). Loss of enteroendocrine cells in autoimmunepolyendocrine-candidiasis-ectodermal-dystrophy (APECED) syndrome with gastrointestinal dysfunction. J Clin Endocrinol Metab.

[16] Montin D, Lala R, Mazza C, Martino S (2008). Atypical onset of APECED (Autoimmune Polyendocrinopathy, Candidiasis, Ectodermal Dystrophy) in two siblings [abstract]. Clin Exp Immunol.

[17] Rodríguez Sánchez De La Blanca A, Loidi Fernández L, Rodríguez Caro R, Roldán Martín MB (2012). Síndrome poliglandular autoinmunitario tipo 1. Diferentes formas de inicio [letter]. Med Clín (Barc).

[18] O’Gorman CS, Schulman R, Lara-Corrales I, Pope E, Marcon M, Grasemann H, Schneider R, Upton J, Sochett EB, Koltin D, Cohen E (2013). A child with autoimmune polyendocrinopathy candidiasis and ectodermal dysplasia treated with immunosuppression: a case report. J Med Case Rep.

[19] Radetti G, Maselli M, Buzi F, Corrias A, Mussa A, Cambiaso P, Salerno M, Cappa M, Baiocchi M, Gastaldi R, Minerba L, Loche S (2012). The natural history of the normal/mild elevated TSH serum levels in children and adolescents with Hashimoto's thyroiditis and isolated hyperthyrotropinaemia: a 3-year follow-up. Clin Endocrinol (Oxf).

[20] Capalbo D, Mazza C, Giordano R, Improda N, Arvat E, Cervato S, Morlin L, Pignata C, Betterle C, Salerno M (2012). Molecular background and genotype-phenotype correlation in autoimmune-polyendocrinopathy-candidiasis-ectodermal-dystrophy patients from Campania and in their relatives. J Endocrinol Invest.

[21] Halonen M, Eskelin P, Myhre AG, Perheentupa J, Husebye ES, Kämpe O, Rorsman F, Peltonen L, Ulmanen I, Partanen J (2002). AIRE mutations and human leukocyte antigen genotypes as determinants of the autoimmune polyendocrinopathy-candidiasisectodermal dystrophy phenotype. J Clin Endocrinol Metab.

[22] Capalbo D, Improda N, Esposito A, De Martino L, Barbieri F, Betterle C, Pignata C, Salerno M (2013). Autoimmune polyendocrinopathy-candidiasis-ectodermal dystrophy from the pediatric perspective. J Endocrinol Invest.

[23] Holzelova E, Vonarbourg C, Stolzenberg MC, Arkwright PD, Selz F, Prieur AM, Blanche S, Bartunkova J, Vilmer E, Fischer A, Le Deist F, Rieux-Laucat F (2004). Autoimmune lymphoproliferative syndrome with somatic Fas mutations. N Engl J Med.

[24] Meloni A, Furcas M, Cetani F, Marcocci C, Falorni A, Perniola R, Pura M, Bøe Wolff AS, Husebye ES, Lilic D, Ryan KR, Gennery AR, Cant AJ, Abinun M, Spickett GP, Arkwright PD, Denning D, Costigan C, Dominguez M, McConnell V, Willcox N, Meager A (2008). Autoantibodies against type I interferons as an additional diagnostic criterion for autoimmune polyendocrine syndrome type I. J Clin Endocrinol Metab.

[25] Husebye ES, Perheentupa J, Rautemaa R, Kampe O (2009). Clinical manifestations and management of patients with autoimmune polyendocrine syndrome type I. J Inter Med.

[26] Scarpa R, Alaggio R, Norberto L, Furmaniak J, Chen S, Smith BR, Masiero S, Morlin L, Plebani M, De Luca F, Salerno MC, Giordano R, Radetti G, Ghizzoni L, Tonini G, Farinati F, Betterle C (2013). Tryptophan hydroxylase autoantibodies as markers of a distinct autoimmune gastrointestinal component of autoimmune polyendocrine syndrome type 1. J Clin Endocrinol Metab.

[27] Betterle C, Volpato M, Rees Smith B, Furmaniak J, Chen S, Zanchetta R, Greggio NA, Pedini B, Boscaro M, Presotto F (1997). II. Adrenal cortex and steroid 21-hydroxylase autoantibodies in children with organ-specific autoimmune diseases: markers of high progression to clinical Addison’s disease. J Clin Endocrinol Metab.

[CR28] The pre-publication history for this paper can be accessed here:http://www.biomedcentral.com/1471-2431/14/272/prepub

